# Evaluation of 5G and Fixed-Satellite Service Earth Station (FSS-ES) Downlink Interference Based on Artificial Neural Network Learning Models (ANN-LMS)

**DOI:** 10.3390/s23136175

**Published:** 2023-07-05

**Authors:** Abdulmajeed Al-Jumaily, Aduwati Sali, Víctor P. Gil Jiménez, Eva Lagunas, Fatin Mohd Ikhsan Natrah, Fernando Pérez Fontán, Yaseein Soubhi Hussein, Mandeep Jit Singh, Fazdliana Samat, Harith Aljumaily, Dhiya Al-Jumeily

**Affiliations:** 1 Wireless and Photonic Networks Research Centre of Excellence (WiPNET), Department of Computer and Communication Systems Engineering, Universiti Putra Malaysia, Serdang 43400, Selangor, Malaysia; 2 Department of Signal Theory and Communications, Universidad Carlos III de Madrid, 28911 Madrid, Spain; vgil@ing.uc3m.es; 3 Interdisciplinary Centre for Security, Reliability and Trust (SnT), University of Luxembourg, L-1855 Luxembourg, Luxembourg; eva.lagunas@uni.lu; 4 Faculty of Agriculture, Universiti Putra Malaysia, Serdang 43400, Selangor, Malaysia; natrah@upm.edu.my; 5 Department of Signal Theory and Communications, Universidad de Vigo, 36310 Vigo, Spain; fpfontan@uvigo.es; 6 Department of Information Systems and Computer Science, Ahmed Bin Mohammed Military College (ABMMC), Doha P.O. Box 22988, Qatar; dr.yaseein@abmmc.edu.qa; 7 Department of Electrical, Electronic and System Engineering, Universiti Kebangsaan Malaysia, Bangi 43600, Selangor, Malaysia; mandeep@ukm.edu.my; 8 Pusat Sains Angkasa Institut Perubahan Iklim, Universiti Kebangsaan Malaysia, Bangi 43600, Selangor, Malaysia; fazdliana@ukm.edu.my; 9 Department of Computer Science and Engineering, Universidad Carlos III de Madrid, 28912 Madrid, Spain; haljumai@inf.uc3m.es; 10 School of Computer Science & Mathematics, Liverpool John Moores University, Liverpool L3 3AF, UK; d.aljumeily@ljmu.ac.uk

**Keywords:** 5G-BS, interference model, FSS Earth station, co-channel and adjacent channel, ANN

## Abstract

Fifth-generation (5G) networks have been deployed alongside fourth-generation networks in high-traffic areas. The most recent 5G mobile communication access technology includes mmWave and sub-6 GHz C-bands. However, 5G signals possibly interfere with existing radio systems because they are using adjacent and co-channel frequencies. Therefore, the minimisation of the interference of 5G with other signals already deployed for other services, such as fixed-satellite service Earth stations (FSS-Ess), is urgently needed. The novelty of this paper is that it addresses issues using measurements from 5G base stations (5G-BS) and FSS-ES, simulation analysis, and prediction modelling based on artificial neural network learning models (ANN-LMs). The ANN-LMs models are used to classify interference events into two classes, namely, adjacent and co-channel interference. In particular, ANN-LMs incorporating the radial basis function neural network (RBFNN) and general regression neural network (GRNN) are implemented. Numerical results considering real measurements carried out in Malaysia show that RBFNN evidences better accuracy with respect to its GRNN counterpart. The outcomes of this work can be exploited in the future as a baseline for coexistence and/or mitigation techniques.

## 1. Introduction

In recent years, the number, variety, and complexity of mobile internet applications have intensively increased. This condition is one of the reasons for developing new fifth-generation (5G) mobile networks. 5G mobile networks are largely expected to be the next important advancement in mobile broadband. A considerable number of countries had already deployed 5G services by 2020. Increased efficiency and performance provides new user information and links to new industries. Recent industries, such as universal surroundings connected to the vast Internet of Things and vehicle communication, have emerged. The industries demand ultralow latency and extreme reliability of 5G mobile communication access. Accordingly, Malaysia has reserved frequencies in the 3.5 GHz range in the C-band and 29.5 GHz in the mmWave band [1].

The 5G 3.5 GHz network is currently in use even in developed countries. However, more bandwidths may be needed with the increase in the volume of mobile traffic. Additional frequency resources are required to meet the demands of 5G. Therefore, the International Telecommunication Union Radio (ITU-R) Regulations designated these frequency resources for the frequency allocation table for the downlink (DL) of the fixed-satellite service Earth station (FSS-ES) and mobile communications services [2,3,4,5].

On this basis, the interference effects-based field test and simulations by using probability or sharing between radio systems operating in co-channel and adjacent channel interferences should be examined to minimise the interference effect and address the limited frequency resources. The Electronic Communications Committee evaluated the existing block edge mask requirements for 5G through an FSS and radar coexistence study conducted at the European Conference of Postal and Telecommunication Administrations. Specific to the range of 3.55 GHz to 3.65 GHz, we examined how 5G base stations (BSs) affect the FSS and ESs. The simulation results based on ITU-R M. 2101 and ITU-R P.452-16 were used to analyse two instances where 5G-BS and FSS-ES interfered with the co-channel and adjacent channels. However, the 5G-BS interference is observed when the DL is considerable. This study used measurements and simulations to design the optimal exclusive zone. The co-channel interference of 5G-BS and FSS-ES was investigated [6,7]. In the literature, Monte Carlo (MC) simulations were used to evaluate the protection distance for 5G-BS and FSS-ES [8,9]. A frequency overlap between 5G-BS and FSS-ES (3.4–3.8 GHz) causes interference.

**The main contributions of this study are as follows:**

1. A 5G model was created using data collected during a measuring campaign in Malaysia.
2. The model’s exclusion zones are based on geographical realities and useful considerations.
3. The paper focuses on minimising excessive interference, lowering designation error in the model, and enhancing comparison rates.
4. In this paper, novel machine learning approaches are used. The radial basis function neural network (RBFNN) and the general regression neural network (GRNN) are used to create multi-objective comparative learning models.
5. The machine learning model has been improved to significantly lessen interference during training.

Future 5G technologies, such as 5G-NR, are intended to improve the capabilities and performance of wireless networks. These advancements may result in the introduction of new features and techniques that alter the interference landscape. 5G-NR, for example, incorporates advanced beamforming and massive Multiple-Input Multiple-Output (MIMO) technologies, which facilitate highly directional and focused transmission. This has the potential to reduce interference to some degree, as the transmission can be more effectively isolated from satellite signals. Future 5G technologies may also include enhanced spectrum management techniques, adaptive modulation and coding schemes, and sophisticated interference cancellation algorithms. These developments can improve spectrum sharing and coexistence with fixed-satellite services (FSS-ES). It is important to note, however, that the specific interference impact of future 5G technologies and the applicability of proposed solutions will depend on their deployment strategies, implementation details, and regulatory frameworks.

As these technologies continue to evolve and mature, it will be necessary to conduct additional research, analysis, and empirical studies in order to assess their interference effects and develop appropriate solutions. While the discussed analysis concentrates on the current state of 5G technology, it is essential to recognise the potential impact of future 5G technologies, such as 5G-NR, on the interference issue. As 5G technology evolves, it will be necessary to conduct ongoing research and implement solutions in order to address any potential new obstacles.

## 2. Materials and Methods

### 2.1. Measurement Scenario of 5G DL

The 5G Base Station (5G-BS) and the fixed-satellite services Earth station (FSS-ES) are using the same frequency, which is the cause of the interference between two channels. The MEASAT-3A satellite with vertical polarisation is used. Co-channel interference, the design of frequency interference, and saturation to satellite ESs are the main problems with the 5G-BS. The total interfering power, location, elevation angle, and other variables of 5G-BS play a key role in the global performance and link availability. This section explains and describes the method for analysing the interference. Part of the 5G-BS signal power component enters the satellite earth frequency band between 3.6 and 4.2 GHz generating interference. A measurement campaign starting on 12 August 2020, at MAEPS Serdang, Universiti Putra Malaysia (UPM), Selangor, Malaysia, was conducted, and data were obtained between 12 and 16 August at the locations to measure the interference caused by a 5G-BS. The experiments were tested based on the 5G-BS parameters, and higher rejection was observed on the first carrier rather than the second carrier of 5G-BS transmissions. Carrier 1 is 3.4–3.5 GHz, and Carrier 2 is 3.5–3.6 GHz. In addition, in the next section, we present the 5G-BS interference model.

### 2.2. 5G-BS Interference Model

**Figure 1:**
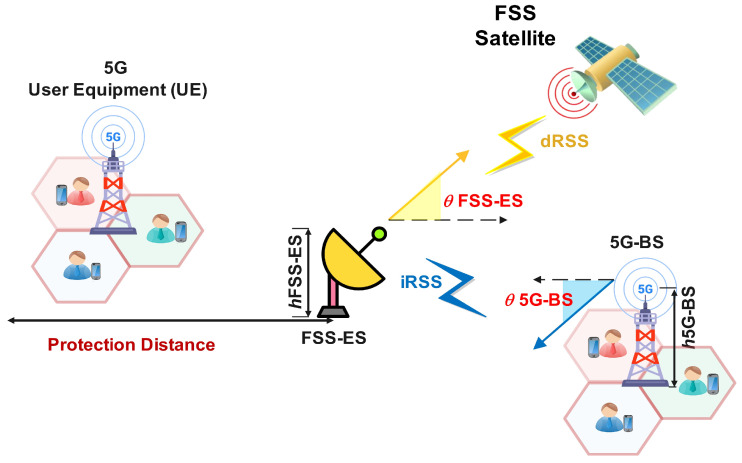
Diagram of 5G-BS interference measurement.

A detailed examination of 5G-BS effect on satellite-received signals are provided in the following analysis, which considers theoretical and experimental evidence. Figure 1 shows the design scheme of the interference model via 5G-BS to FSS-ES. The desired received signal power DL from the satellite at FSS-ES is denoted by dRSS. The DL-received signal power from the 5G-BS at the user equipment (UE) is denoted by iRSS. The elevation angle of the received signal of the BS is denoted by θ5G-BS, and the received signal of FSS-ES is θFSS-ES. The height of the BS is h5G-BS, and the height of FSS-ES is hFSS-ES, as shown in Figure 1. The worst case is when the 5G-BS is using sectorial antennas because it generates interference in the whole area. The main parameters for 5G-BS are summarised in Table 1. The 5G-BS DL interferes with satellite receivers due to C-band spectrum sharing. We suppose that 5G-BS is covering a particular area. Sector antennas are considered for the 5G-BS to guarantee the worst-case interference model scenario.

The bandwidth required to support 5G requirements in terms of capacity and latency is 100 MHz [10] at frequencies below 6 GHz. Table 2 depicts the three 5G-BS carrier frequencies assigned to a sampling frequency (frequency 2 in the first spectrum block, frequency 1 in the second spectrum block, and frequency 3 in the third spectrum block). One 5G-BS zone may have different equipment from different operators, and each carrier frequency is considered to operate in at least one of these zones. The extracted C-band low-noise block (LNB) with a bandpass filter (BPF), the C-band LNB with BPF, and the new C-band LNB with BPF are used. A guard band (GB) of 100 and 50 MHz is used for 5G-BS. After being idle near an FSS-ES for a few minutes, the 5G-BS mobile device started downloading the full buffer to check the effect of interference. The direct-facing FSS-ES and the indirect-facing FSS-ES are 85 m between the 5G-BS and FSS-ES [10].

### 2.3. FSS-ES Interference Model

**Figure 2:**
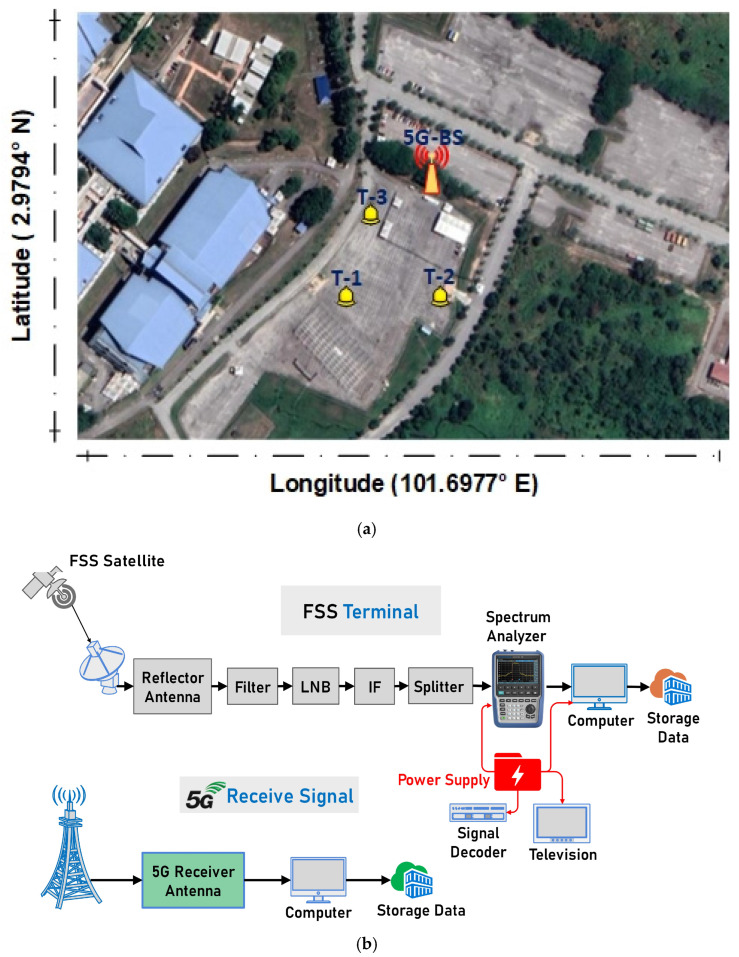
Schematic of FSS-ES interference measurement; (**a**) The measurements Location; (**b**) The FSS-ESS and 5G-BS test diagram.

Test 1 (T-1), Test 2 (T-2), and Test 3 (T-3) in Figure 2a represent the FS-ES experiments’ tests locations of the coexistence model between 5G-BS and FSS-ES. The main parameters for FSS-ES are summarised in Table 3. The 5G DL transmission interferes with an FSS-ES receiver operating in the 3.625 GHz to 4.2 GHz range, as shown in Figure 2b. Therefore, we established the coexistence of 5G-BS and FSS-ES with 85 m distance as an interference source. Several studies, such as [11,12], have mentioned that C-band communications are seriously affected. Therefore, we installed filters on FSS-ES to determine if the interference coexistence of 5G-BS and FSS-ES affected the performance, and we addressed the interference coexistence. Thus, the 5G-BS can be used near an FSS-ES at an 85 m distance. The 5G-BS and FSS-ES enhance the interference for satellite signal isolation. In order to keep the FSS-ES at a safe distance and prevent interference from the 5G-BS, we must find a way to reduce the interference signals. They can use spatial attenuation or medium isolation and antenna directivity to further reduce the interference. When the 5G-BS and its receiving antenna are configured to have their peak amplitudes pointing in different directions “direct-facing VSAT and then indirect”, the satellite signal can be better isolated from the 5G interference, and the interference is reduced. The transmission power of the satellite uplink (UL) station and the power of the RF signal are increased to help mitigate the interference to a certain degree. Increased satellite DL signal strength helps reduce the 5G-BS transmission power whilst improving the satellite signal-to-noise ratio and the 5G interference-to-noise ratio.

This section examines the specific effects of 5G interference on fixed-satellite services (FSS-ES). It offers a comprehensive analysis of the interference model and parameters described in Section 2.2, 5G-BS Interference Model, and Section 2.3, FSS-ES Interference Model. The paragraph discusses the use of sector antennas for 5G base stations (5G-BS) in order to generate the most severe interference scenario possible. The required bandwidth for 5G and the designated carrier frequencies for the 5G-BS are described. The paragraph also describes the experiments conducted to test the compatibility of 5G-BS and FSS-ES, as well as the installation of interference-reducing filters. For interference reduction, spatial attenuation, medium isolation, and antenna directivity are suggested. Boosting the transmission capacity of satellite uplink stations and the strength of the satellite downlink signal can also help mitigate 5G interference.

### 2.4. SEAMCAT Simulation

**Figure 3:**
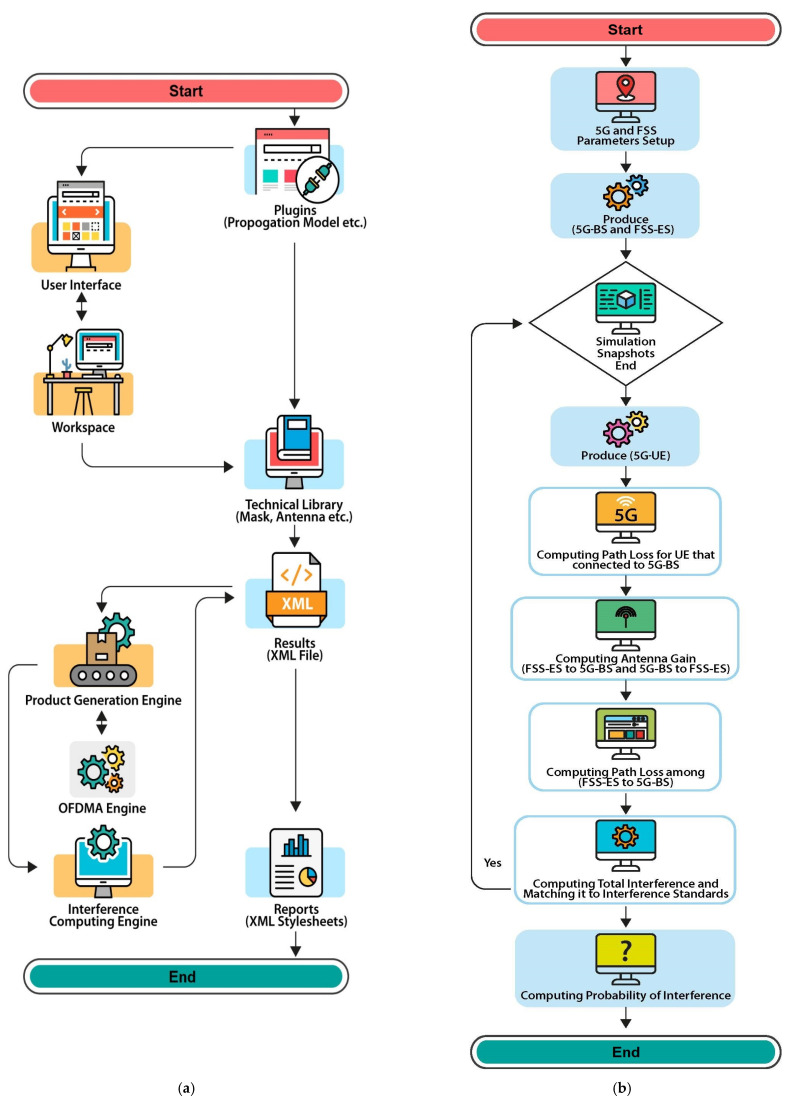
SEAMCATs and interference modelling. (**a**) SEAMCAT main fundaments parts; (**b**) Interference algorithm.

The European Communications Office provides SEAMCAT [13,14], an interference analysis method based on the Monte Carlo (MC) approach to analyse and complete the measurements with simulations. This method can analyse the coexistence of FSS-ES operating in adjacent frequency bands by statistically simulating the radio interference. Figure 3a,b show the basic structure of SEAMCAT [15]. Users can select an interference situation in the workspace. Each snapshot evaluates the interference for the received signal strength (RSS) and the one created by the activity creation system. Compared with the RSS, interference via the protection threshold determines whether an interference signal arises and explains the process in more detail.

#### 2.4.1. Simulation of Interference Model

As shown in Figure 1, the interference scenario where the effectiveness of FSS-ES might be seriously affected by a practical 5G deployment is evaluated to analyse and determine the effect of interference from 5G-BS on FSS-ES. The 5G-BS is assumed to be deployed symmetrically around the circle’s circumference through a radius matching protection distance via the FSS-ES centre distance. The number of 5G-BS is computed by using Equation (1) [16].

(1) $${\mathrm{ND}}_{BS}=\frac{2\pi d_{p}}{0.8}$$

where *ND_BS_* refers to the number of deployed BSs. The protection distance is denoted by *d_p_* (km) and concentrated on two types of interference: co-channel and adjacent channel interferences. In other words, each sector has one specific UE. The aggregate interference of all 5G-BSs is then computed by using Equation (2). However, the received signal power DL from the 5G-BS into UE is iRSS.

(2) $$I_{aggregated}=10\log\sum_{n=1}^{n=ND_{BS}}10^{iRSS_{n}/10}$$

#### 2.4.2. Simulation Parameters

##### 5G-BS

The 5G network is a powerful system using time-division duplexing, and the UL and DL use the same frequency. In this study, 5G-BS interfering with DL connectivity is analysed. However, the same conclusions can be easily extrapolated to UL because they use the same frequency, albeit with less transmit power. The simulation specifications and parameters for the 5G-BS are described in Table 1 [17,18]. The 3.4–3.8 GHz, −53 dBm and 100 MHz values were the operative frequencies, transmission powers, and bandwidths, respectively. The azimuth signalling for each antenna was distributed randomly [19] to cope with all the possibilities. Beamforming is a critical part of 5G-BS specialised capabilities. SEAMCAT’s antenna library includes an ITU-R M.2101-0 recommendation model, which is used to create a composite antenna design.

**Figure 4:**
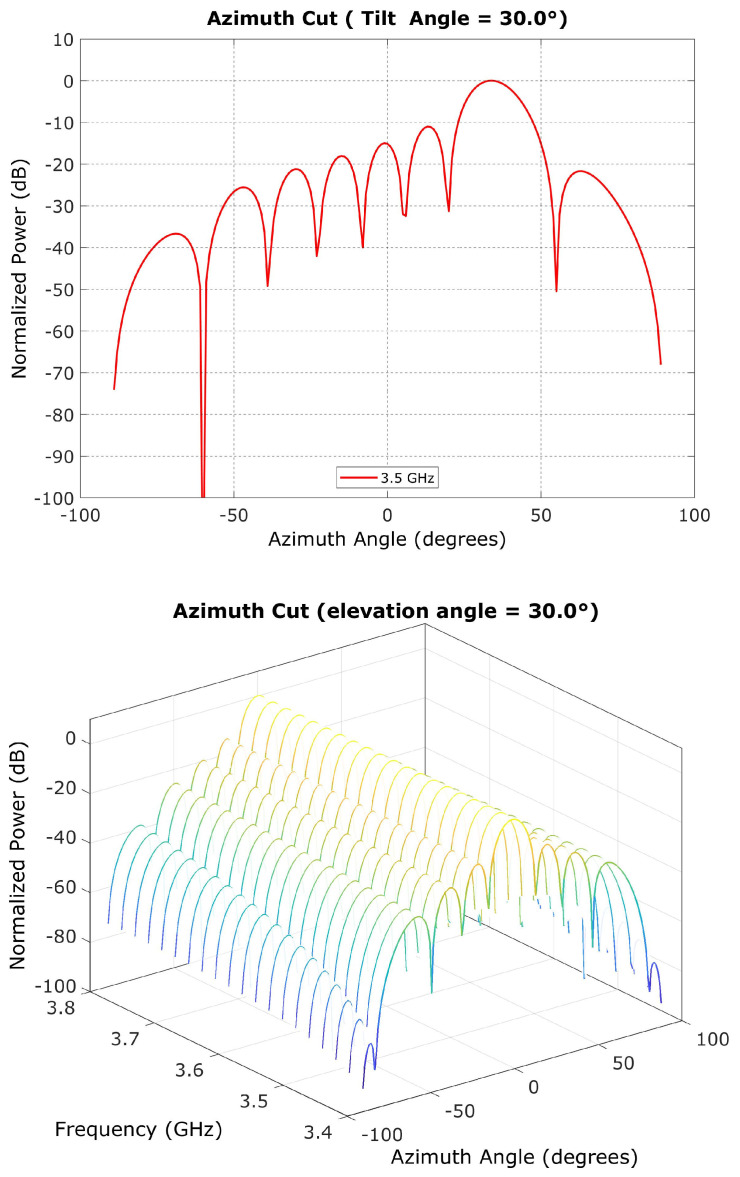
Antenna gain as a function of antenna azimuth angle (°).

Each antenna boresight was constantly aimed at the UE, and each antenna’s mechanical elevation pointing was fixed to 15° for simplification [20,21]. The antenna azimuths based on the antenna characteristics listed in Table 1 are shown in Figure 4. On the basis of azimuth angle, the combined antenna peak gain for the 8 × 8 antennas with 5 dBi of each peak gain was 25 dBi [22,23].

**Figure 5:**
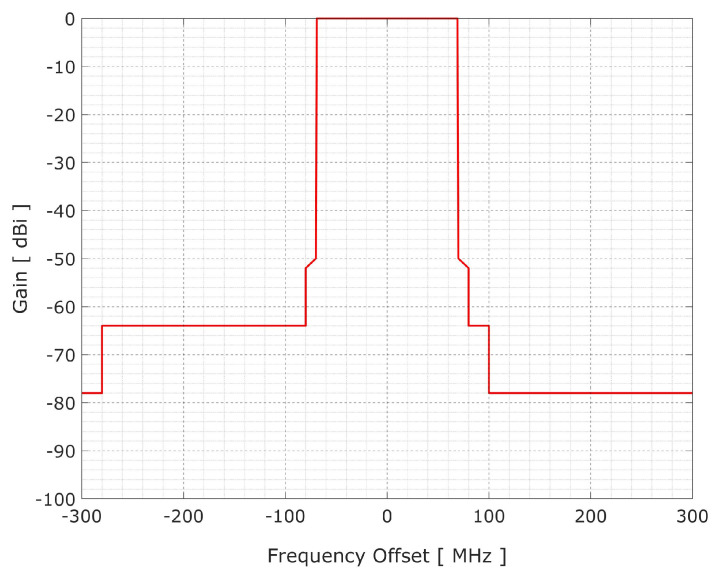
Spectrum emission masks of 5G-BS transmitters.

The ITU-R P.452-16 propagation model was used to compute the propagation loss from 5G-BS to FSS-ES. Figure 5 depicts a 5G-BS spectrum emission mask for detecting out-of-band and spurious emissions in the adjacent interference. Table 4 describes the 5G-BS out-of-band and spurious emissions masks [24].

##### FSS-ES

A geostationary satellite and an ES on the surface communicating through the FSS-ES were modelled on the basis of Table 2. The target was an FSS-ES because it was more susceptible to the interference of 5G-BS DL signals than the satellite [25] due to the lower transmit power at the UL. Co-channel interference with 5G-BS occurred at 3.4–3.8 GHz, whereas adjacent interference occurred at 3.9 GHz. The 100 MHz bandwidth was selected for the analysis, with a noise generation “noise bandwidth” of 5 dB. The noise floor value was determined to be −125 dBm. The −12.2 dB is the threshold noise level of the interference-to-noise ratio (INR) for evaluating the interference. Elevation was slanted by 30° on the mechanical pointer. However, the horizontal orientation remained consistent towards the satellite. The FSS-ES antenna gain pattern was based on ITU-R S.2196 [26]. The radiation patterns can be divided into two types. We chose a pattern that can be implemented in the case when *D*/*λ* is more than 54.5. This pattern can be inferred by using Equations (3)–(6).

(3) $$G(\varphi )=G{\left(\frac{D}{\lambda }\varphi \right)}^{2},\;m_{max}$$

(4) $$G\left(\varphi \right)=\left\{\begin{array}{l} G_{1}\leq \varphi_{m}\leq \varphi_{r};\\ 32-25\log\varphi_{r}\times 114{\left(\frac{D}{\lambda }\right)}^{-1.09}\\ -10\leq 48^{\circ }\leq \varphi \leq 180^{\circ } \end{array}\right.,\varphi_{r}\leq \varphi \leq 48^{\circ }$$

(5) $$\varphi_{m}=\sqrt[{\frac{20\lambda }{D}}]{{G1}_{max}}$$

(6) $$\varphi_{r}=15.85{\left(\frac{D}{\lambda }\right)}^{-0.6}$$

where *G_*1*max_* is the antenna maximum gain, *D* is the antenna diameter, *λ* is the wavelength, and *φ* is the angle used to determine the antenna performance. The vertical gain of the FSS-ES antenna by utilising the antenna specifications and calculations from Table 2 is shown in Figure 2a. The antenna gain for the horizontal and vertical planes is equivalent. Therefore, a maximum antenna gains of 58 dBi was achieved in the horizontal and vertical directions.

### 2.5. Artificial Neural Network Learning Model (ANN-LM)

**Figure 6:**
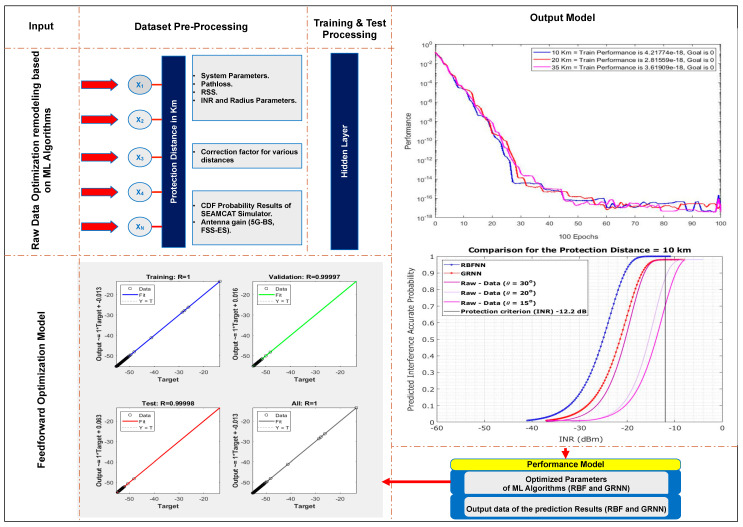
ANN-LM schematic.

The paper employs ANN-LMs to improve modelling outcomes. ANN-LMs are potent AI models capable of learning from data, recognising patterns, and making accurate predictions with minimal human intervention if adapted and described appropriately. The iterative nature of ANN-LMs enables them to adapt to newly introduced data. A robust neural network model with multiple layers that exceed the number of layers in conventional neural networks is utilised in this study. In the context of ANN, use layers to model particular patterns, such as basic edges or object parts, which are then passed to subsequent layers. The configuration of radial basis function neural network (RBFNN) and generalized regression neural network (GRNN) models can be provided. To address data issues, preprocessing techniques were employed, and the data were separated into training and testing sets. The choice of ANN-LM structure and network validation training took data classification and measurement into account. The fifth phase involved a method based on selection, which resulted in the effective creation of a network model with high assessment precision. Figure 6 depicts the use of RBFNN and GRNN ANN-LMs, both of which are endowed with pruning capabilities, to predict the output of the system. In addition, a model-based predictive optimisation strategy was used to calculate the protection distance between 5G-BS and FSS-ES techniques. As shown in Figure 6, six input features were used for model predictions in this paper. These inputs include specific data points associated with 30-degree, 20-degree, and 15-degree angles. As a safeguard against interference, the interference-to-noise ratio (INR) was also included. In addition, pertinent 5G and fixed-satellite services (FSS) parameters were evaluated. These parameters collectively serve as the basis for our prediction models, facilitating accurate predictions based on the supplied data and parameters.

Initially, we chose the radial basis function neural network (RBFNN) and the generalized regression neural network (GRNN) as our machine learning algorithms for assessing downlink interference in the context of 5G and the FSS Earth station (FSS-ES). These models were chosen due to their demonstrated efficacy in dealing with nonlinear relationships and their successful application in telecommunications and interference analysis. In addition, RBFNN and GRNN provide efficient training and prediction procedures, which are essential to our research. Regarding simulation parameters, we took industry standards and previous research into cautious consideration. In our simulation, a heterogeneous network consisting of multiple 5G base stations and FSS Earth stations was deployed in an urban setting. To assure realistic simulations, the number and placement of these stations were determined based on actual deployment scenarios. In addition, we included various channel characteristics, such as path loss, fading, and shadowing, based on empirical models and measurements specific to the investigated frequency range.

This allowed us to mimic the wireless propagation environment accurately and capture realistic interference scenarios. The significant impact it has on propagation characteristics, interference levels, and the difficulties of coexistence between 5G and FSS-ES systems influenced the choice of the frequency range for our study. The sub-6 GHz frequency band was the primary focus of our research. By examining the frequency range where potential interference issues between 5G and FSS-ES may arise, our research seeks to provide insights and solutions for interference management in these systems. Traditional techniques include frequency planning, power management, and antenna design. Support vector machines (SVM), interference cancellation, accuracy is frequently employed machine learning algorithms in this context. Within the domain of artificial neural network language models (ANN-LMs), RBFNN and GRNN were evaluated. A feed-forward neural network comprised of neurons with radial basis functions is adept at approximating complex nonlinear mappings. GRNN is a memory-based algorithm that excels at pattern recognition and regression. Real-world datasets and interference mitigation-specific performance metrics, such as the interference-to-signal ratio (ISR) or bit error rate (BER), are necessary for evaluating the effectiveness of these approaches. Comparative studies and experiments reveal the strengths and weaknesses of various techniques. In Table 5, we can see the proposed ANN-LMs alongside three other methods: SVM and interference cancellation. Accuracy, computational complexity, scalability, and adaptability are only some of the criteria that are compared across the columns. The actual figures are based on the efficiency and features of each technique in inquiry.

The precise number of hidden layers and nodes in the hidden layer is not specified in our paper. The configuration of radial basis function neural network (RBFNN) and generalized regression neural network (GRNN) models can be provided.

#### 2.5.1. Radial Basis Function Neural Network (RBFNN)

**Figure 7:**
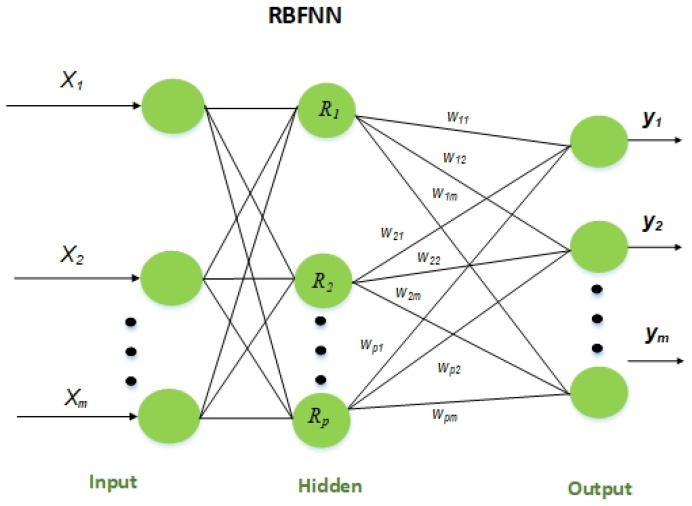
Conceptual framework for RBFNN topology.

The RBFNN network stands out for its simple construction, quick training and learning unity, and accurate approximation of nonlinear processes. It is a proactive network with three layers and one hidden layer. The m input nodes, P hidden neuron layers, and n output layers of the RBFNN neural network are shown in Figure 7.

The following formula, ${X=[x_{1},x_{2}\ldots x_{m}]}^{T}\in R^{n}$, denoted the data input while deriving the following formula, ${Y=[y_{1},y_{2}\ldots y_{m}]}^{T}$, indicated the data output. This is apart from the radial basis utility of the layer hidden using the regular gaussian utility, the response output. Radial basis functions (RBFs), which have radial symmetry, are the activation functions utilised as basic functions in the network’s hidden layer [29]. It is possible to express the widely used Gaussian function as Equation (7).

(7) $$R_{i}\left(x\right)=exp\left(-\frac{{\left\|x-C_{i}\right\|}^{2}}{2\sigma_{i}^{2}}\right),i=1,2,\ldots ,P$$

In the above network, the activation function of the hidden layer is represented by $R_{i}(x)$ for the input $x_{i}$, and the centre is designated as $c_{i}$ of the *i*-basis function. As stated in [30], the network’s hidden and output layers have a linear relationship, whereas the input and hidden layers display a nonlinear relationship. Equation (8) can be used to represent the network’s output.

(8) $$y_{i}=\sum_{i=1}^{P}\omega_{ki}R_{i}\left(x\right),k=1,2,\ldots ,q$$

where q is the number of output layer nodes, and $\omega_{ki}$ is the adjustment weight across the output and hidden layers.

#### 2.5.2. General Regression Neural Network (GRNN)

**Figure 8:**
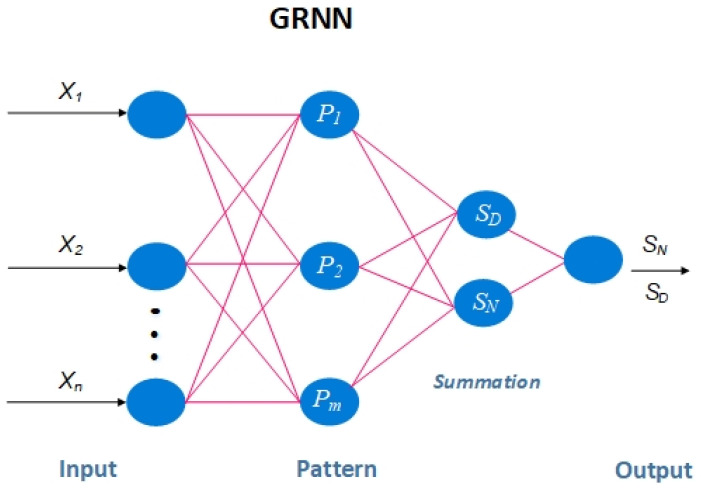
Conceptual framework for GRNN topology.

The hidden layer of the single pass neural network type has a Gaussian activation function. Layers for input, hiding, summation, and division are intended to be present. The model’s prediction performance is improved by the fact that only one smoothing factor parameter needs to be changed during model creation. Equation (9) and Figure 8 illustrate how the theory behind GRNN is based on nonlinear regression analysis.

(9) $$\hat{Y}=E(\frac{y}{x})=\frac{\int_{-\infty }^{\infty }yf(X,y)dy}{\int_{-\infty }^{\infty }f(X,y)dy}$$

where *x* and *y* represent the random variable measurements. Probability distribution in its entirety is represented by the function f(x,y). Y is the result of deriving from the input data and finding a prediction to be $\hat{Y}$.

Equation (10) represents the normal distribution as the following:

(10) $$\hat{f}=(X,y)=\frac{1}{n(2\pi {)}^{\frac{p+1}{2}}\sigma^{p+1}}\sum_{i=l}^{n}exp\left[-\frac{\left(x-X_{i}{)}^{T}(x-X_{i}\right)}{2\sigma^{2}}\right]exp\left[-\frac{(x-Y_{i}{)}^{2}}{2\sigma^{2}}\right]$$

The observations for samples *x* and *y* are denoted by $X_{i}$ and $Y_{i}$, respectively, where X is the model input variable, n is the sample size, and p is a random set variable, while x is the number of dimensions of σ, the smooth factor of the model, and f(x,y) and replace with f(x,y) in Equation (10), resulting in Equation (11).

(11) $$\hat{Y}(X)=\frac{\sum_{i=l}^{n}Y_{i}exp\left[\frac{\left(X-X_{i}{)}^{T}(X-X_{i}\right)}{2\sigma^{2}}\right]}{\sum_{i=l}^{n}exp\left[\frac{\left(X-X_{i}{)}^{T}(X-X_{i}\right)}{2\sigma^{2}}\right]}$$

There are four layers in total in the GRNN network structure: Source nodes comprise an input layer. The input variables are sent straight from the input layer through number of neurons that meet the value of the input *X_i_* to the next layer of patterns. With the next transfer efficiency as Equation (12), layer number neurons then relate to the training sample number of *n*, and each neuron represents a single sample.

(12) $$P_{i}exp\left[\frac{\left(X-X_{i}{)}^{T}(X-X_{i}\right)}{2\sigma^{2}}\right]\;i=1,2,\ldots ,n$$

The weighted sum $S_{Nj}$ and the sum of the numbers $S_{D}$, which respective transfer functions are depicted in Equations (13) and (14), are used to sum the layer in the process of accumulating layers.

(13) $$S_{D}=\sum_{i=l}^{n}P_{i}$$

(14) $$S_{Nj}=\sum_{i=l}^{n}y_{ij}\;P_{i}$$

where i is a neuron’s transfer function and $P_{i}$ is a portion of the pattern layer. As seen in the following Equation (15), the symbol $y_{ij}$ represents the pattern layer section j of an epoch with a summation layer.

(15) $$y_{i}=\frac{S_{Nj}}{S_{D}}$$

## 3. Results

### 3.1. Measurements

The results from the FSS-ES field test at 85 m distance are shown in Table 6, Table 7, Table 8 and Table 9. The ping loss is 0%, and the bit error rate (BER) is 10^−6^. No BER was obtained, although three carriers were considered in testing the transmitted power (TX) of 5G-BS and were reduced to 100 W. However, the received signal frequency for the new C-band LNB without filter (3.705 GHz), C-band LNB with filters (3836.27 GHz), and extended C-band with several filters (3.705 GHz) were all tested. Also, 5G carrier 1 (full load) and carrier 2 (full download) operated at 100 MHz GB. Carrier 1 (100% load), carrier 2 (100% load), carrier 3 (full download), and the 5G-BS were on at all times. A difference was observed between RF interference (RFI) and RFI^*1^, whereas RFI^*1^ denotes that the link passes but with a high BER.

#### 3.1.1. Broadband Tuner

**Figure 9:**
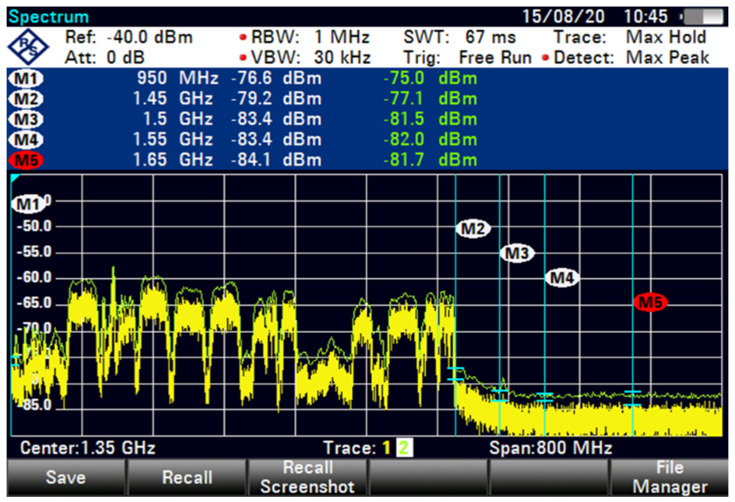
FSS for 85 m (new C-band OFF).

The MEASAT-3A satellite with vertical polarisation is swept, and the result is depicted in Figure 9. The FSS-ES with the phrase new C-band OFF at an 85 m distance denotes the signal intensity under clear sky conditions without GB, which is around −81.7 dBm. The DL frequency of a C-band satellite is typically between 3.625 and 4.2 GHz, and the downconversion frequency of LNB ranges from 950 MHz to 1.65 GHz. The operator’s 5G frequency allotment is between 3.4 and 3.5 GHz, whereas the frequency resources received by the Malaysian Communications and Multimedia Commission are between 3.5 and 3.6 GHz. The two frequencies are close to the C-band satellite signal frequency. When the signal travels through the LNB, the downconversion frequencies are 1.450 and 1.65 GHz.

**Figure 10:**
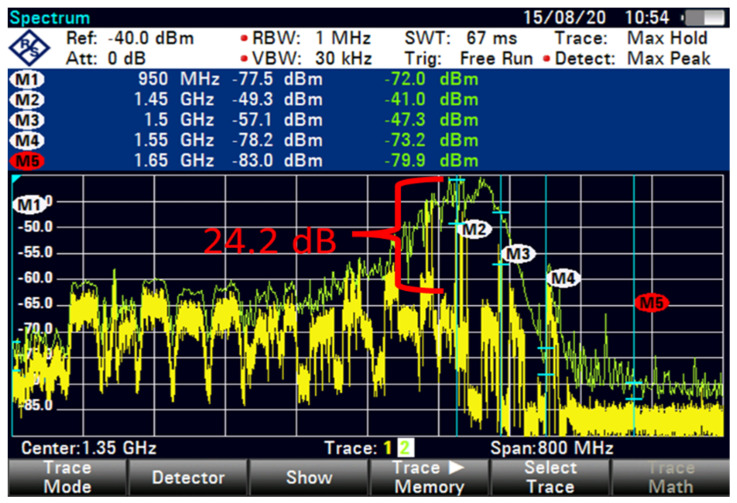
FSS for 85 m (new C-band 100 MHz GB).

As shown in Figure 10, the FSS-ES with the term new C-band 100 GHz GB at an 85 m distance denotes an interference signal exceeding 24.2 dB, and the signal intensity is around −79.9 dBm. However, the downconversion frequency of LNB ranges from 950 MHz to 1.65 GHz. The operator’s 5G frequency allotment is between 3.4 and 3.5 GHz, whereas the frequency resources received by the Malaysian Communications and Multimedia Commission are between 3.5 and 3.6 GHz. The two frequencies are close to the C-band satellite signal frequency. When the signal travels through the LNB, the downconversion frequencies are 1.450 and 1.65 GHz.

**Figure 11:**
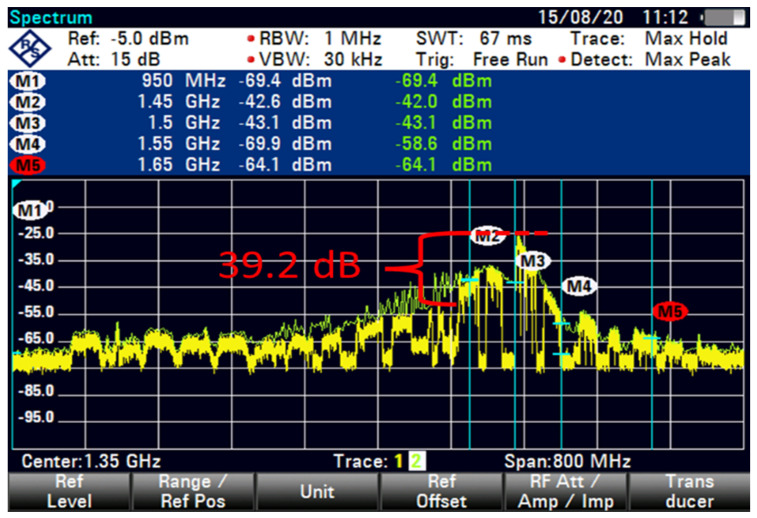
FSS for 85 m (new C-band 50 MHz GB).

As shown in Figure 11, the FSS-ES with the term new C-band 50 GHz GB at an 85 m distance results in an interference signal exceeding 39.2 dB, and the signal intensity is around −64.1 dBm. However, the downconversion frequency of LNB ranges from 950 MHz to 1.65 GHz. The operator’s 5G frequency allotment is between 3.4 and 3.5 GHz, whereas the frequency resources received by the Malaysian Communications and Multimedia Commission are between 3.5 and 3.6 GHz. The two frequencies are close to the C-band satellite signal frequency. When the signal travels through the LNB, the downconversion frequencies are 1.450 and 1.65 GHz. The affected C-band satellite signals can be temporarily removed to resume broadcasting immediately. The FSS-ES blocked the interference because its LNB cannot fully reject or reduce the 5G signals to levels below which the satellite can receive them.

#### 3.1.2. Broadband Tuner with Filter

**Figure 12:**
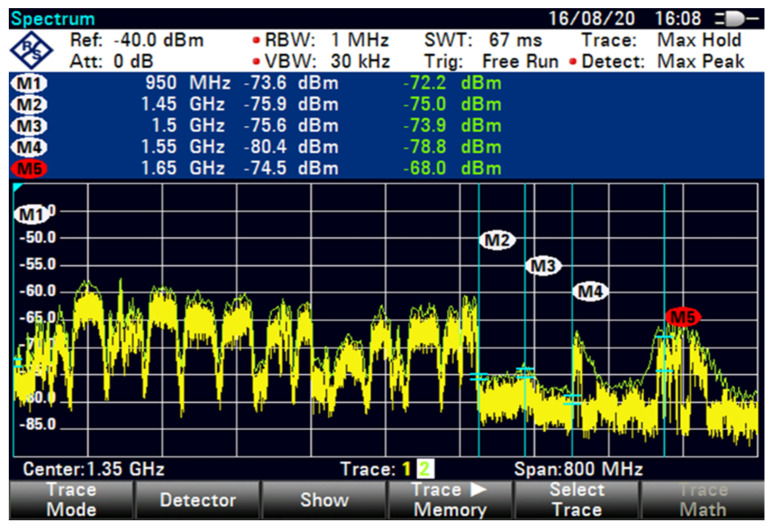
FSS for 85 m (extended C-Band + Filter-1, 100 MHz GB).

**Figure 13:**
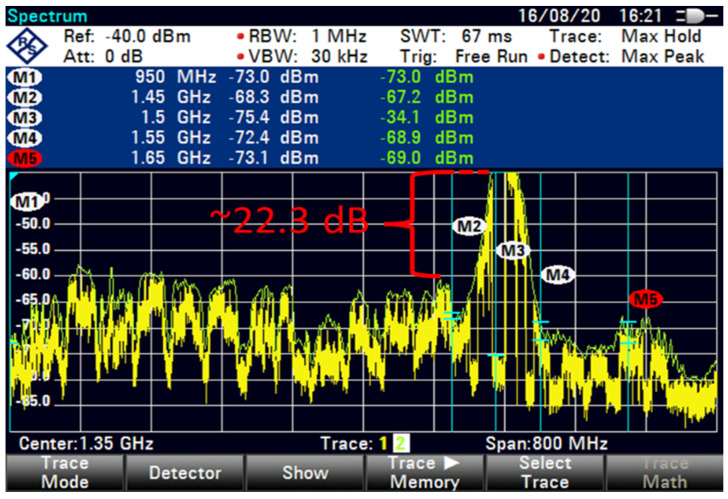
FSS for 85 m (extended C-Band + Filter-1, 50 MHz GB).

**Figure 14:**
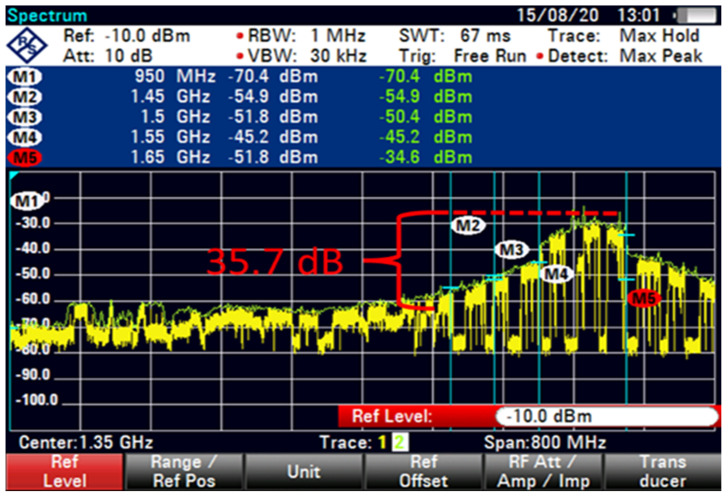
FSS for 85 m (extended C-Band + Filter-2, 100 MHz GB).

The FSS-ES used a filter technique for the term extended C-band in adding filter-1 with 100 MHz GB and low interference at an 85 m distance, as shown in Figure 12. A similar filter technique with 100 MHz GB exhibited a minimal increased interference with 22 dB, as shown in Figure 13. However, the filter technique for the extended C-band in adding filter-2 with 100 MHz GB showed differences in the shape of interference that reduced the interference by using a different filter technique with 35.7 dB, as shown in Figure 14.

**Figure 15:**
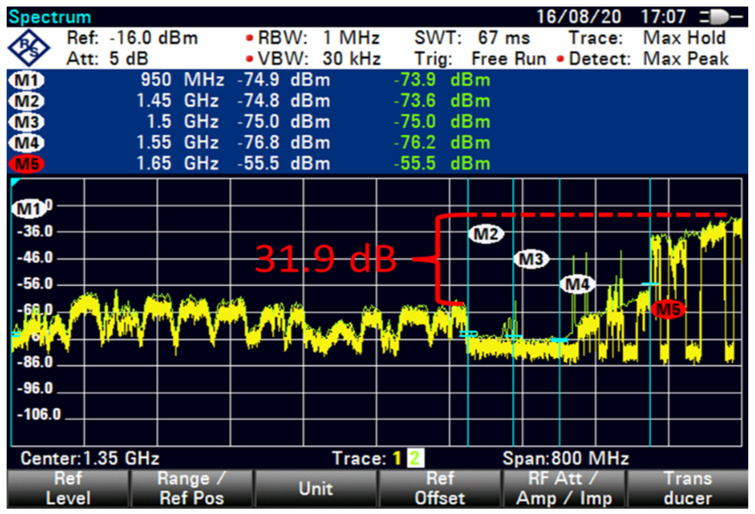
FSS for 85 m (extended C-Band + Filter-5 100 MHz GB no download).

The interference was reduced to 31.9 dB by using filter 5, as shown in Figure 15. At higher FSS DL frequencies, the level of LNB blocking interference decreased exponentially (T-1). The LNB frequency range did not play an essential role in reducing the interference effect because no interference was observed at 85 m with 100 MHz GB for all types of LNB when paired with BPF with sufficient rejection. At 85 m distance or more, no remarkable effect was found on direct-facing FSS-ES because no interference was observed with 100 MHz GB, as shown in Figure 9, Figure 10, Figure 11, Figure 12, Figure 13, Figure 14 and Figure 15. The results are shown in Table 6, Table 7, Table 8 and Table 9.

Table 8 shows the test matrix: direct-facing VSAT and then indirect at 85 m. Extended C-Band-LNB + BPF, C-Band-LNB + BPF, and new C-Band-LNB + BPF, 5G off, then 100 MHz Gard Band (GB), and lastly, 50 MHz GB. The 5G cellular was on idle near VSAT and then performed a full buffer download. And the frequency range was C-Band-LNB: 3625 to 4200 MHz; extended C-Band LNB: 3400 to 4200 MHz; and the new C-Band LNB: 3700 to 4200 MHz (with built-in BPF).

### 3.2. Simulation Results

**Figure 16:**
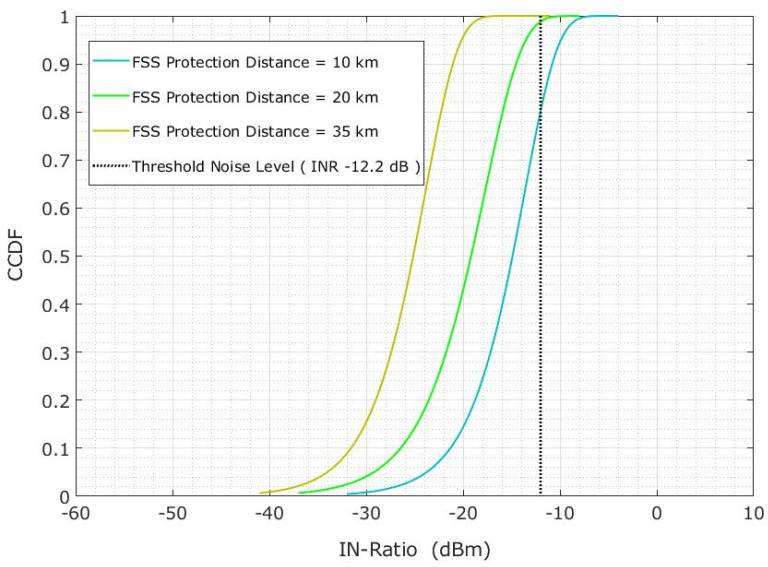
Co-channel interference scenario for the INR CDF is evaluated on the basis of the protection distance.

The system parameters mentioned in this section were used in the MC simulations of 5000 snapshots for each scenario. Table 3 depicts the FSS-ES parameters. An interference-to-noise ratio (INR) of −12.2 dB was chosen as the threshold noise level for the specified parameters. Figure 16 shows the INR cumulative distribution function (CDF) at 15° elevation angle via 5G-BS in co-channel interference. The vertical black line is the threshold noise level and is used to define the interference. Each plotline indicates a CDF for 10, 20 and 35 km protection distances, and the probability of having an INR lower than −12.2 dB is 39.1%, 62.8%, and 97.4%, respectively. Therefore, Equation (16) is used to calculate the result. However, most radio communication techniques should have a similar level of reliability, which should be more than 95%. The corrected interference of less than 5% should be acceptable.

(16) $$P_{1}=1-P\left(INR<-12.2\right)$$

A correlation was found between the 5G-BS elevation and the interference probability, so further investigation was conducted. Let us suppose an INR lower than −12.2 dB, where the probability ‘P INR > −12.2 dB’ is higher and should maintain the interference probability below 5%. Thus, a minimum distance of 35 km is needed to maintain the INR of −12.2 dB, as shown in Figure 16. Significant changes were observed on the CDF plotlines, which were more than the INR value of −12.2 for 10, 20, and 35 km at the 5G-BS elevation angles of 15°, 20°, and 30°, as shown in Figure 16.

**Figure 17:**
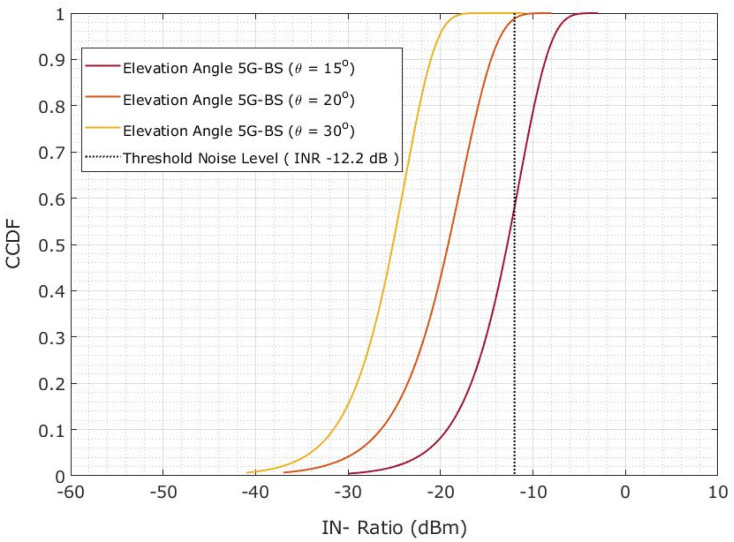
Co-channel interference scenario for the INR CDF is evaluated for 5G-BS elevation angle based on the protection distance of 10 km.

The CDF is shown in Figure 17 when the protection distance is 10 km. However, the plotlines show that the CDF changes the INR for 5G-BS at a 15° elevation angle. The plotline illustrates the CDF difference over INR at 20° and 30° elevation angles of the 5G-BS. However, the estimated probability is significant at 69% and 97.6% when the 5G-BS elevation angles are 20° and 30°, respectively. The elevation angles of 5G-BS are increased from 15° to 20° and from 0° to 30°. The 29.8% and 27.3% increase in the probability of achieving an INR of less than −12.2 dB was observed. The interference probability is decreased by 27.4% and 29.9%.

**Figure 18:**
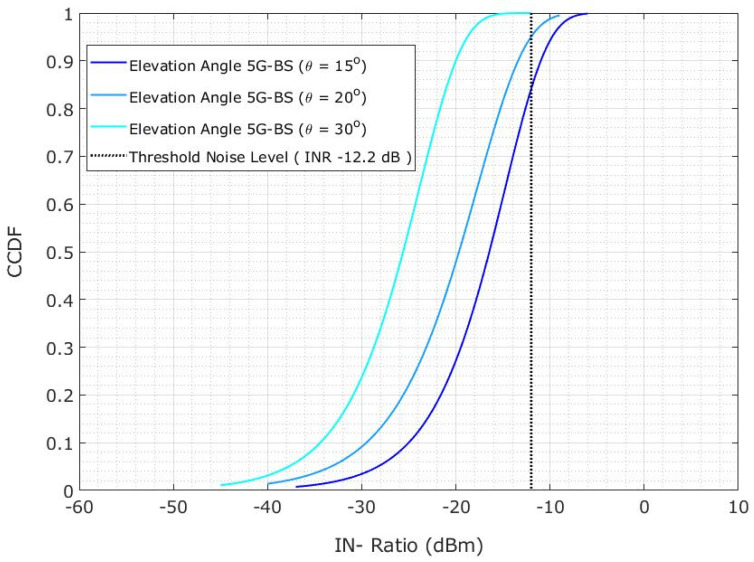
Co-channel interference scenario for the INR CDF is evaluated for 5G-BS elevation angle based on the protection distance of 20 km.

Figure 18 shows the CDF probability for a 20 km protection distance. The plotline represents the CDF variation across the INR for a 5G-BS elevation angle of 15°. With the 5G-BS elevation angles of 20° and 30°, the CDF variation across the INR is shown in the plotlines. The CDF across the INR changes depending on the 5G-BS elevation angles of 20° and 30° within the protection distance of 20 km. However, the changing of the 5G-BS elevation angle may relieve the limitation on the protection distance if the protection distance cannot be maintained effectively.

**Figure 19:**
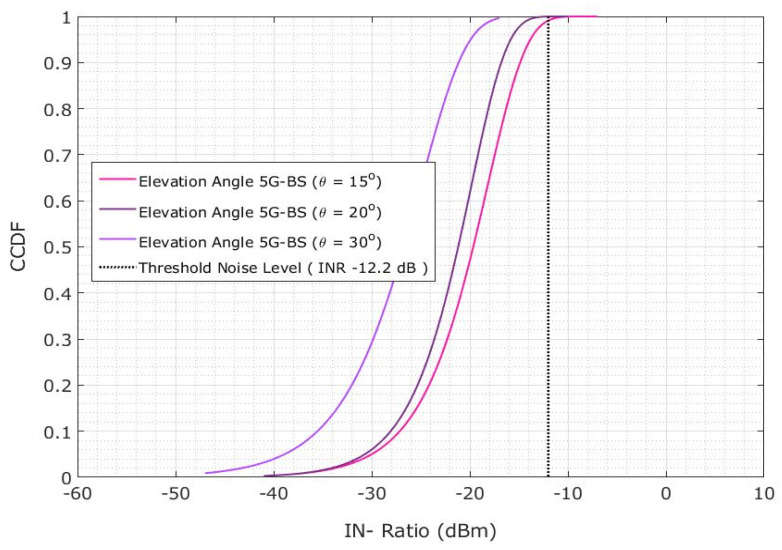
Co-channel interference scenario for the INR CDF is evaluated for 5G-BS elevation angle based on the protection distance of 35 km.

As shown in Figure 19, the interference probability lowered by 59.5% when the elevation angle of 5G-BS was adjusted from 15° to 30° at a 35 km protection distance [31]. However, the interference probability decreased by only 20.4% at a protective distance of 35 km. The interference probability increases with the decrease in protection distance due to the difference in the elevation angle of 5G-BS [32].

**Figure 20:**
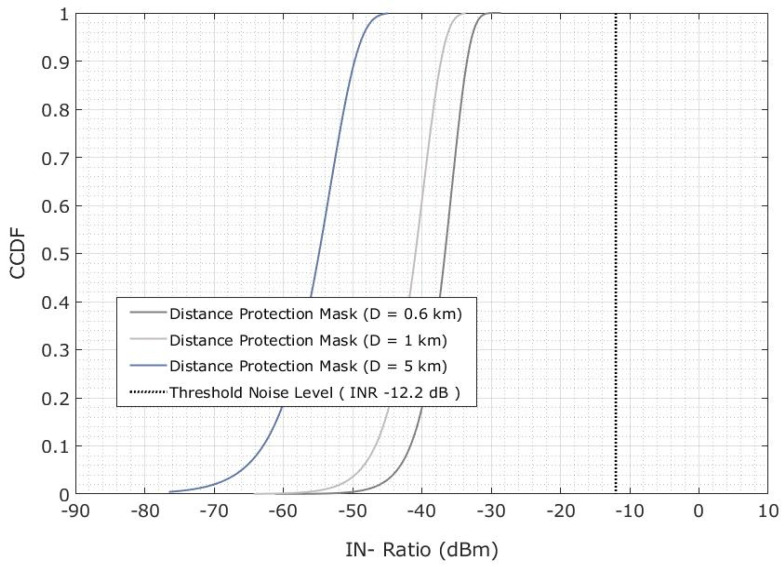
Adjacent interference scenario for the INR CDF is evaluated on the basis of the protection distance.

The CDF of adjacent interference probability is shown in Figure 20 and is obtained by using the spectrum emission mask for a protection distance of 0.6 km. The additional values are addressed in Table 10, where the cells did not extend beyond. The INR of −12.2 dB is the number of interferences detected at a protective distance of 0.6 km. An adjacent interference assessment is shown in Figure 20. Therefore, the FSS-ES is not affected in the 5G-BS because the spectrum emission mask features are lower than the adjacent interference.

**Figure 21:**
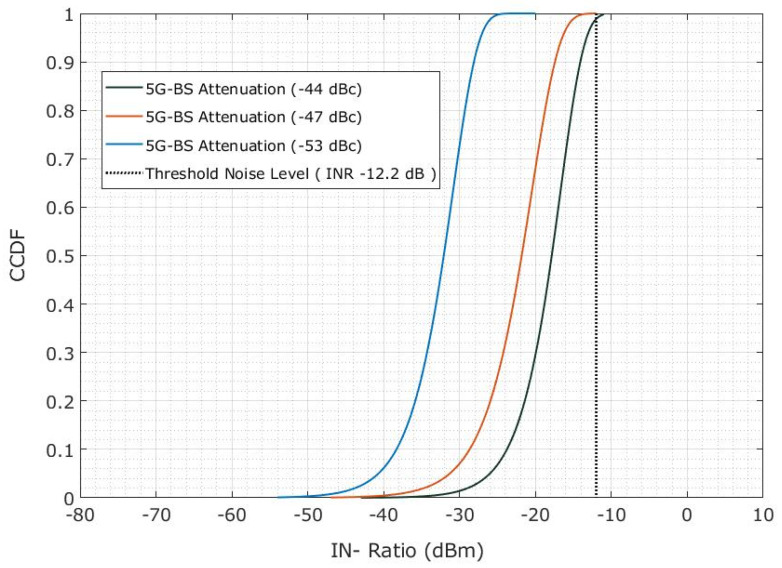
Attenuation CDF for the INR of adjacent interference is based on the out-of-band emission mask of 5G-BS.

We focused on determining the out-of-band emission mask level causing interference in the FSS-ES by adjusting the protection distance of 5G-BS to 0.6 km [33]. Figure 21 depicts the INR CDF as a function of the adjacent channel’s attenuation level by using the 5G-BS out-of-band emission mask. Therefore, the INR probability lower than −12.2 dBm will be 100% if the attenuation is higher than −53 dBc, indicating no interference. Interference occurred when the INR was less than −12.2 dBm (−55 dBc), and the attenuation was smaller than that. Therefore, attenuation of −53 dBc had a 98.6% chance of satisfying an INR of less than −12.2 dBm. Thus, attenuation of at least −53 dBc was needed to achieve an interference probability of less than 5%.

### 3.3. ANN-LM Results

**Figure 22:**
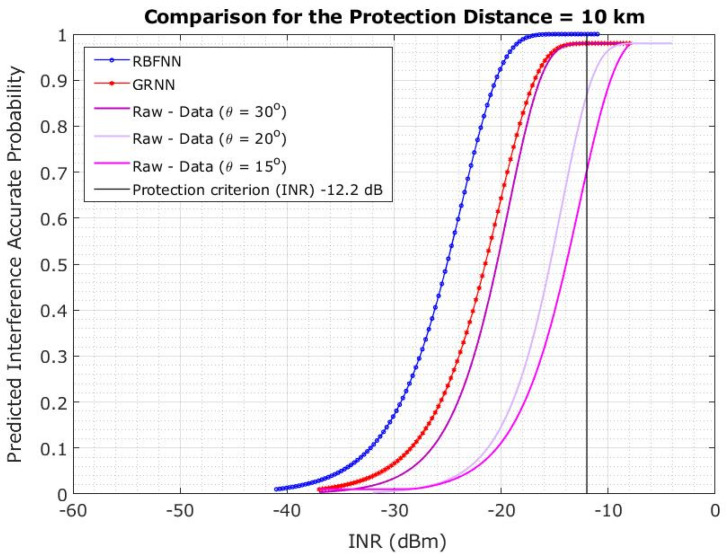
Predicted interference probability using ANN-LM RBFNN and GRNN for protection distance of 10 km.

**Figure 23:**
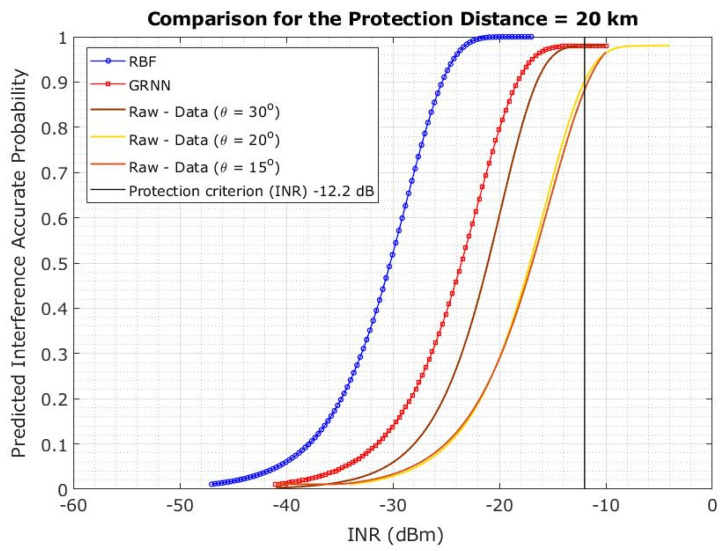
Predicted interference probability using ANN-LM RBFNN and GRNN for protection distance of 20 km.

**Figure 24:**
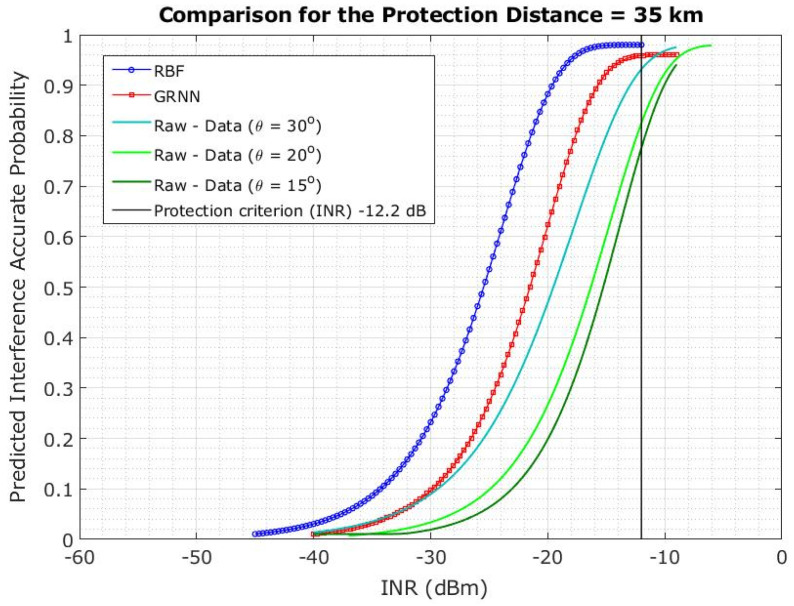
Predicted interference probability using ANN-LM RBFNN and GRNN for protection distance of 35 km.

The results showed that the RBFNN-predicted models outperformed the GRNN models in terms of protection distance and elevation angle. The prediction model based on RBFNN showed 100% accuracy. Thus, the curve closer to the desired INR of −12.2 dB is more effective in blocking the interference. The ANN-LM considered elevation angles of 15°, 20°, and 30° for protection distances of 10, 20, and 35 km, respectively. Therefore, the ANN-LMs (RBFNN and GRNN) can be used to accurately predict the interference at various protection distances, as shown in Figure 22, Figure 23 and Figure 24.

### 3.4. ANN-LM Optimisation Performance Modelling Results

**Figure 25:**
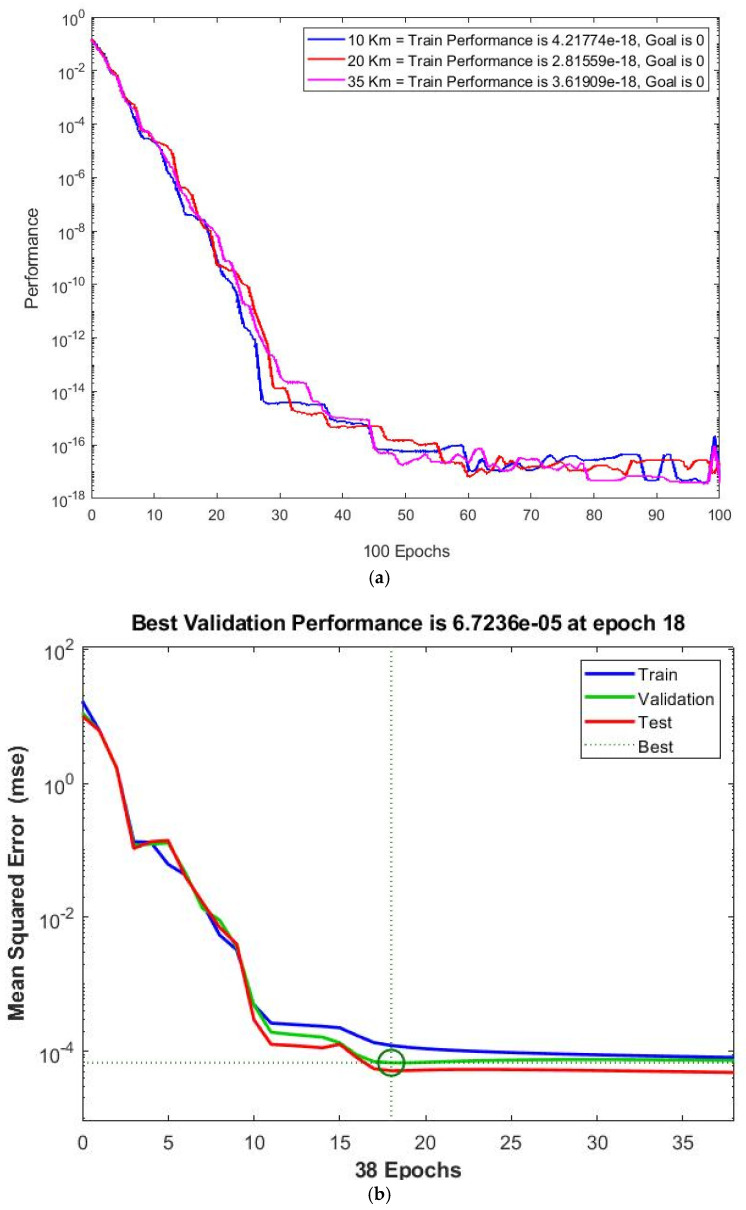

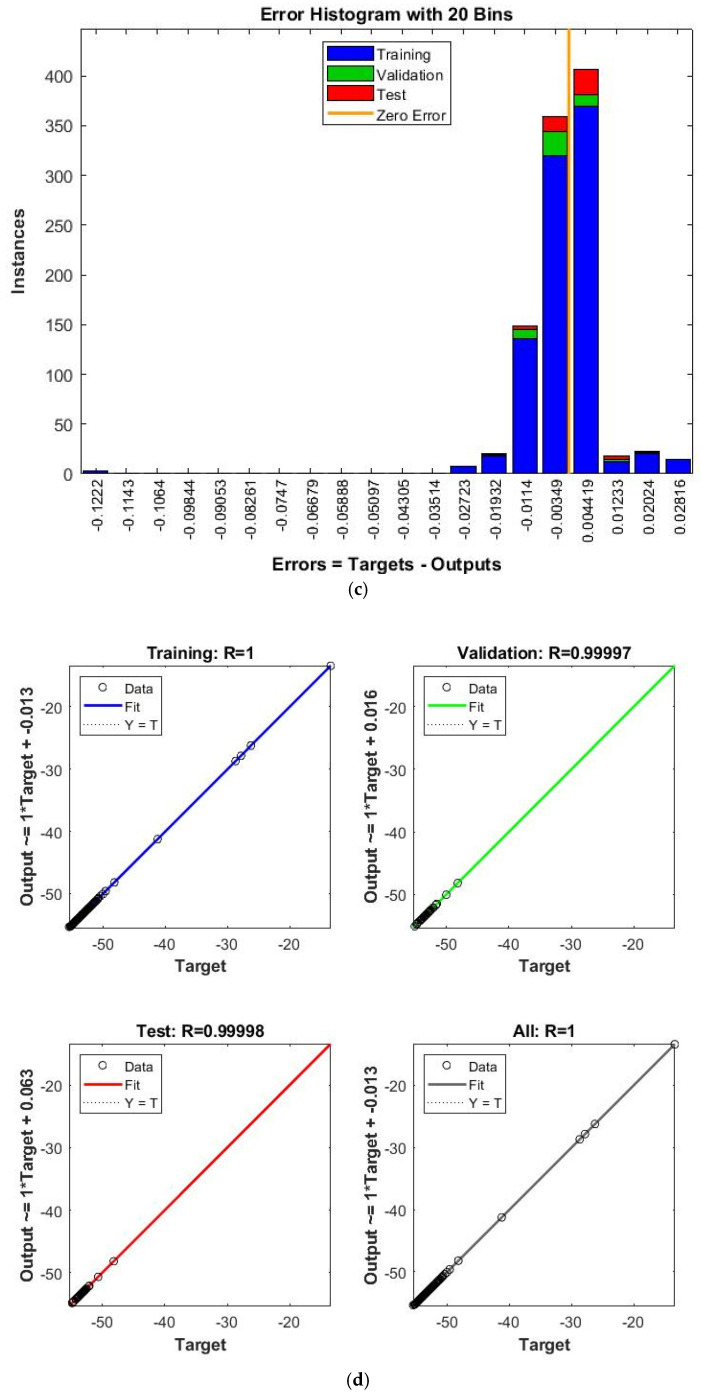
ANN-LM validation and performance technique for protection distance between 5G-BS and FSS-ES. (**a**) ANN-LM performance training vs. epochs of three protection distances 10, 20, and 35 km. (**b**) Mean squared error performance. (**c**) Error histogram instances. (**d**) Particular correlation coefficient.

This section demonstrates the effect of interfering signals on the distance between 5G-BS and FSS-ES in detail. The network algorithm, training function, transfer function, number of hidden layers, number of neurons in the hidden layer and epochs, dataset separation, and performance combinations are examined through ANN-LM optimisation performance modelling. ANN-LM algorithms are tested for overfitting by comparing the performance of the training and testing algorithms. Figure 25a–d illustrate the effectiveness of ANN-LM optimisation performance modelling. As shown in Figure 25a, the performance is similar regardless of the protection distance. This finding is interesting because it makes the performance independent of the protection distance. Figure 25a shows the training process for RBFNN and GRNN algorithms, which mostly reach the design goal of zero error. The significant training performance is approximately 4.21774 × 10^−18^ for RBFNN and GRNN, with 100 training epochs for a protection distance of 10 km. The models trained using RBF and GRNN algorithms mostly achieve the design goal error of zero with training epochs of 100, and the significant training performance is approximately 2.81559 × 10^−17^ for 20 km. The models trained using the RBFNN and GRNN algorithms achieve a near-zero design goal error with 100 training epochs, and the significant training performance is approximately 3.61909 × 10^−18^ for 35 km. The RBFNN and GRNN models are trained symmetrically until reaching 100 epochs of the same data.

The training results of ANN-LM optimisation performance at different protection distances are shown in Figure 25b. Training, testing, and validation plotlines were all noticeable. The training, validation, testing, and best performance are represented by a blue curve, a green curve, a red curve, and a dotted line, respectively. The number of training epochs and the mean squared error (MSE) are plotted on the x and y axes, respectively. Epoch 18 appeared when the validation performance dipped below acceptable levels, and the training was stopped. The error histogram plot is shown in Figure 25c. The error histogram plot shows the difference between the critical and predicted values. The regression coefficient R measures the correlation between the outputs and the goals. As shown in Figure 25d, the regression coefficient between the outputs and targets measures how well the targets explain the variation in the outputs. A regression coefficient of R = 1 was found during data training, implying that the training was perfect.

A statistically significant difference was observed between the regression coefficients of 0.99997 and 0.99998 for data validation and testing, respectively. Hidden neurons in the network had a correlation coefficient (R) of 38 epochs between the expected output and the target it provides. A correlation coefficient of R = 1 was observed, indicating that the present model perfectly reproduced the expression levels of interference in the protection distance between 5G-BS and FSS-ES. The intention during data training and testing is to maintain the average MSE at as low a level as possible. Epoch neurons range from 0 to 100, and the MSE outputs were calculated. The maximum and average errors are evaluated on the basis of the neuron output values, as shown in Table 11.

Table 11 presents MSE values for the RBFNN and GRNN algorithms. The RBFNN algorithm mean squared error (MSE) values for various protection distances (in kilometres) and numbers of neurons are shown in Table 11. The RBFNN technique produced an MSE value of 0.168253 at a protection distance of 10 km when zero neurons were used (0 neurons). The MSE value decreased considerably to 1.08857 × 10^−15^ when there were 50 neurons added. The MSE value was further decreased to 4.17612 × 10^−18^ after the number of neurons was increased to 100. This shows the processing by the RBFNN algorithm to produce the MSE values for the specified protection distances and neuronal counts. These data show that by lowering the MSE and producing more precise predictions, increasing the RBFNN algorithm’s neuron count can greatly enhance its performance.

A further indication that the RBFNN algorithm can handle the data effectively and deliver results efficiently. The GRNN algorithm produced an MSE value of 0.168258 for a protection distance of 10 km. When there were 50 and 100 neurons, the MSE values fell considerably to 1.08860 × 10^−15^ and 5.17622 × 10^−18^, respectively. This shows the processing of the GRNN algorithm to compute the MSE values for the given protection distances and neuronal counts. These findings show that increasing the number of neurons in the GRNN algorithm can significantly enhance its performance, as shown by the decline in MSE values. The GRNN algorithm is reasonably efficient in processing the data and generating results. The MSE value at a protection distance of 20 km for the RBFNN algorithm was 0.128807 when no neurons (0 neurons) were used.

The MSE values dropped to extremely low levels, measuring 4.8190 × 10^−15^ and 2.81559 × 10^−17^, respectively, as the number of neurons reached 50 and 100. This decrease in MSE indicates that the RBFNN algorithm’s ability to accurately capture underlying patterns is enhanced by adding more neurons. However, when no neurons were used, the GRNN algorithm at a 20-kilometre protection distance produced an MSE value of 0.138813. The MSE values were 4.8211 × 10^−15^ and 7.74620 × 10^−18^, respectively, for 50 and 100 neurons. According to these findings, the GRNN algorithm, despite having somewhat higher MSE values than the RBFNN algorithm, performs similarly in terms of prediction errors.

Both the RBFNN and GRNN algorithms show their capacity to obtain low MSE values at a protection distance of 20 km, with variations depending on the number of neurons used. In terms of MSE values, the RBFNN algorithm typically beats the GRNN algorithm. The MSE value at a protection distance of 35 km for the RBFNN algorithm was 0.168153 when no neurons (0 neurons) were used. The MSE values considerably dropped to 1.88574 × 10^−13^ and 3.61909 × 10^−18^ with 50 and 100 neurons, respectively. As the number of neurons rises, the MSE decreases, indicating increased performance and accuracy. At a protection distance of 35 km, the GRNN algorithm, on the other hand, generated an MSE value of 0.168270 when no neurons were used. The MSE values for 50 and 100 neurons were slightly higher, at 1.89594 × 10^−13^ and 7.44741 × 10^−18^, respectively.

## 4. Discussion

The results from the FSS-ES field test at an 85 m distance depicted the loss as 0%, and the bit error rate (BER) is 10^−6^. No BER was obtained, although three carriers were considered in testing the transmitted power (TX) of 5G-BS and were reduced to 100 W. However, the received signal frequency for the new C-band LNB without filter (3.705 GHz), C-band LNB with filters (3836.27 GHz), and extended C-band with several filters (3.705 GHz) were all tested. The 5G carrier 1 (full load) and carrier 2 (full download) operated at 100 MHz GB. Carrier 1 (100% load), carrier 2 (100% load), carrier 3 (full download), and the 5G-BS were on at all times. A difference was observed between RF interference (RFI) and RFI*1, whereas FRI*1 denotes that the link passes but with a high BER.

The interference is caused by the operator’s 5G frequency allotment on the C-band satellite signal frequency. The FSS-ES measurements show that without a 5G signal, the signal intensity of the C-band satellite signal is around −81.7 dBm at an 85 m distance. However, when the 5G signal with 100 GHz GB is present, the interference signal exceeds 24.2 dB, and the signal intensity is around −79.9 dBm. It is important to note that the downconversion frequencies of the LNB range from 950 MHz to 1.65 GHz, and the 5G frequency allotment is between 3.4 and 3.5 GHz, which are close to the C-band satellite signal frequency. This could cause interference and affect the quality of the satellite signal.

The broadband tuner is experiencing interference from the 5G signals in the operator’s frequency allotment range, which is close to the C-band satellite signal frequency. When the signal travels through the LNB, the downconversion frequencies are 1.450 and 1.65 GHz, which may be causing the interference. The FSS-ES is able to block some of the interference caused by the 5G signals, but it is not able to fully reject or reduce the 5G signals to levels below which the satellite can receive them. As a result, the affected C-band satellite signals may need to be temporarily removed in order to resume broadcasting immediately.

It appears that the FSS-ES successfully reduced interference using a filter technique for the extended C-band signals. Filter-1 with 100 MHz GB and low interference resulted in a minimal interference increase of 22 dB, while filter-2 with 100 MHz GB reduced the interference by 35.7 dB. Filter-5 reduced the interference to 31.9 dB. The LNB frequency range did not play a significant role in reducing the interference effect, as no interference was observed with 100 MHz GB for all types of LNB when paired with BPF with sufficient rejection. At a distance of 85 m or more, there was no remarkable effect on direct-facing FSS-ES, as no interference was observed with 100 MHz GB.

The conducted Monte Carlo simulations of 5000 snapshots were performed for each scenario, with the FSS-ES parameters at an INR threshold of −12.2 dB to define the interference level and the INR cumulative distribution function (CDF) at a 15° elevation angle via 5G-BS in co-channel interference. We found that a minimum distance of 35 km is needed to maintain the INR of −12.2 dB, and significant changes were observed on the CDF plotlines for different elevation angles. The authors also investigated the effect of protection distance on the interference probability, the CDF probability for a 20 km protection distance. Also, we found that changing the elevation angle of 5G-BS may relieve the limitation on the protection distance if the protection distance cannot be maintained effectively.

Furthermore, the authors investigated the adjacent interference probability using the spectrum emission mask for a protection distance of 0.6 km. They found that the FSS-ES is not affected by the 5G-BS because the spectrum emission mask features are lower than the adjacent interference. Finally, the authors determined the out-of-band emission mask level causing interference in the FSS-ES by adjusting the protection distance of 5G-BS to 0.6 km. They found that an attenuation of at least −53 dBc was needed to achieve an interference probability of less than 5%.

The results of the study indicate that the RBFNN-predicted models performed better than the GRNN models in terms of protection distance and elevation angle. Specifically, the RBFNN model achieved 100% accuracy in predicting interference, and the curve closer to the desired INR of −12.2 dB was found to be more effective in blocking interference. The ANN-LMs, including both RBFNN and GRNN, were able to predict interference at various protection distances accurately, and that performance is independent of protection distance.

The training performance for both RBFNN and GRNN algorithms was found to be significant, with both models mostly achieving the design goal error of zero with training epochs of 100. Specifically, the significant training performance for RBFNN was approximately 4.17612 × 10^−18^ for a protection distance of 10 km, 2.81559 × 10^−17^ for 20 km, and 3.61909 × 10^−18^ for 35 km. The RBFNN and GRNN models were trained symmetrically until reaching 100 epochs of the same data. These findings suggest that the RBFNN algorithm is a promising approach for accurately predicting interference in wireless communication systems.

The paper used ANN-LM algorithms to present an interference analysis between 5G base stations and FSS Earth stations in the 3.4–4.2 GHz frequency band. The results showed that the RBFNN-predicted models outperformed the GRNN models in terms of protection distance and elevation angle. The RBFNN model showed 100% accuracy, and the curve closer to the desired INR of −12.2 dB was found to be more effective in blocking interference. The ANN-LMs (RBFNN and GRNN) can accurately predict the interference at various protection distances, regardless of the protection distance.

The ANN-LM optimisation performance modelling was also carried out to examine in detail the effect of interfering signals on the distance between 5G-BS and FSS-ES. The training results of ANN-LM optimisation performance showed that the present model perfectly reproduced the expression levels of interference in the protection distance between 5G-BS and FSS-ES. The average MSE was maintained at as low a level as possible, and the maximum and average errors were evaluated based on the neuron output values.

Finally, the study provided valuable insights into the interference analysis between 5G base stations and FSS Earth stations in the 3.4–4.2 GHz frequency band using ANN-LM algorithms. The RBFNN-predicted models outperformed the GRNN models, and the ANN-LMs (RBFNN and GRNN) can be used to accurately predict the interference at various protection distances. The ANN-LM optimisation performance modelling also provided detailed insights into the effect of interfering signals on the distance between 5G-BS and FSS-ES.

## 5. Conclusions

This study investigated the interference between new 5G DL and FSS-ES signals in the frequency range of 3.4–4.2 GHz. Measurements and MC simulations were used for the SEAMCAT interference analysis. No adjacent interference was recorded between the 5G-BS and the FSS-ES because the transmission power level dropped due to filters and masks, which is extremely convenient. Co-channel interference necessitates a protection distance of at least 35 km to work properly. However, the protection distance can be reduced by increasing the elevation angle of 5G-BS. This finding is especially interesting because it allows some scenarios in which the protection distance can be reduced (if no other option is available). Interference models between 5G-BS and FSS-ES involving GBs, antenna configuration, and FSS-ES bandwidths were optimised for potential deployment. Coexistence issues between 5G-BS and FSS-ES can benefit from these interference analysis methods and results. The coexistence effect may be approximated up to an unlimited frequency band distance by using the co-channel interference model. An ANN-LM interference model was presented. An innovative interference model between FSS-ES and 5G-BS based on different distances with highly accurate ANN-LM prediction algorithms and small effort was developed. This interference might be used to deploy and implement 5G-NR and satellite communications. Machine learning can be applied to previously simulated data in new contexts or at other frequencies in the future. The design and development of sophisticated analysis processes can be performed with the assistance of ANNs. Furthermore, to increase comparability and broaden our understanding of interference mitigation techniques in 5G networks, future research should consider incorporating data from a variety of geographical regions to account for regional deployment and environmental differences. It is essential to evaluate a variety of 5G deployment scenarios and the influence of environmental factors on interference patterns. To facilitate such comparisons, standard evaluation metrics should be developed. In addition, longitudinal studies can shed light on performance over the long term. By adhering to these recommendations, researchers can obtain a comprehensive understanding of the efficacy and adaptability of proposed techniques across various network environments and geographic locations.

## Figures and Tables

**Table 1:** 5G-BS parameters.

Parameters	Value
Frequency Supported range	3400~3800 MHz
Bandwidth	50~100 MHz
5G-BS carriers’ frequency:	3400~3500 MHz; 3500~3600 MHz; 3600~3650 MHz
Antenna Gain	25 dBi
Transmitter power (Tx)	53 dBm (200 W)
Antenna	One sector (80° N) with 3 Active Antenna Units (AAUs)
Mechanical tilt	15°
Site height above mean sea level (AMSL)	~30 m
Antenna height AMSL	~35 m
EIRP	78 dBm/100 MHz
OOB emission	−13 to −15 dBm/100 MHz

**Table 2:** 5G-BS DL spectrum allocation parameters.

Carrier Frequency	Guard Band—100 MHz	Guard Band—50 MHz
T-1	3.4–3.5 GHz	3.4–3.5 GHz
T-2	3.5–3.6 GHz	3.5–3.6 GHz
T-3	3.6–3.650 GHz	3.6–3.650 GHz

**Table 3:** FSS-ES DL spectrum allocation parameters.

Parameters		Value	
Frequency range		3705 MHz (Horizontal)	
Bandwidth		6 MHz	
Antenna Gain		37.5 dBi	
Antenna diameter		2.4 m	
FSS-ES	Extended C-Band LNB + BPF	C-Band LNB + BPF	Unit
Blocking level	−60	−60	dBm
OOB/spurious level	−124.9	−124.9	dBm/MHz
Filter rejection	55	82.5	dB at 3.6 GHz
	54.8	54.8	dB at 3.650 GHz

**Table 4:** Unwanted emission limits for broadband wireless access (BWA).

Frequency—GHz	Emission Limits BWA	Bandwidth/MHz
Unwanted emission (EIRP)	−62 dBW/MHz	100
Unwanted emission with filter (antenna port)	−89 dBW/MHz	100

**Table 5:** The proposed ANN-LMs alongside three other methods.

Refences	Method	Computational Complexity	Scalability	Accuracy (%)	Flexibility
[27]	Interference Cancellation	High	Low	82.3	High
[28]	Random Forest	Moderate	Moderate	92.7	High
[28]	Support Vector Machines	High	Moderate	89.5	Low
Proposed ANN-LMs		Low	High	100	Moderate

**Table 6:** FSS-ES field test results for 85 m (T1 and T3).

Test No.	FSS-ES	Facing	Elev. Angle	LNB + BPF	Rx Freq. (GHz)	100 MHz GB	50 MHz GB
T-1	MESSAGE	Direct	77°	New C-Band only	3.705	RFI	RFI
T-3	MESSAGE	Direct	77°	Extended C-Band with Filter 1	3.705	OK	RFI
T-3	MESSAGE	Direct	77°	Extended C-Band with Filter 2	3.705	RFI	RFI
T-3	MESSAGE	Direct	77°	Extended C-Band with Filter 5	3.705	RFI	RFI

**Table 7:** FSS-ES field test results for 85 m (T2).

Test No.	FSS-ES	Facing	Elev. Angle	LNB + BPF	Rx Freq. (GHz)	100 MHz GB	50 MHz GB
T-2	MESSAGE	Direct	77°	C-band with Filter 5	3836.27	RFI	RFI
T-2	MESSAGE	Direct	77°	Extended C-Band with Filter 5	3836.27	RFI	RFI^*1^
T-2	MESSAGE	Direct	77°	Extended C-Band with Filter 1 and 5	3836.27	OK	RFI

**Table 8:** FSS-ES field rest results for 85 m BPF and GB used.

Extended C-Band LNB + BPF GB 100 MHz							C-Band LNB + BPF—GB 50 MHz				
No	Zone	5G-BS Height (m)	Distance (m)	FSS-ES	Angle (°)	Block. Level @3.6 GHz/dBm	Block. Level @3.650 GHz/dBm	Block. Level @3.6 GHz/dBm	Block. Level @3.650 GHz/dBm	OOB Level dBm	RFI
1	UPM	31	85	MESSAGE	77°	−71.97	−71.77	−99.47	−71.77	−82.97	Yes
2	UPM	31	80	MESSAGE	77°	−69.48	−69.28	−96.98	−69.28	−80.48	Yes
3	UPM	31	50	MESSAGE	77°	−65.58	−65.38	−93.08	−65.38	−76.58	Yes
4	UPM	31	20	MESSAGE	77°	−52.58	−52.38	−80.08	−52.38	−63.58	Yes

**Table 9:** FSS-ES field test results for 85 m BFF filters used.

Filter No	Model No	Frequency MHz	S21-dB	S11-dB	S22-dB	Delay (ns)	100 MHz or 50 MHz GB
1	BPF-3700S	3600	−68	-	-	-	100 MHz
		3650	−10	-	-	-	
		3700	−0.4	−31	−25	8.9	
		4000	-	-	-	4.5	
		4200	−0.5	−25	−21	8.7	
2	13961W	3600	−44	-	-	-	50 MHz
		3650	−49	-	-	-	
		3700	−1.2	−24	−21.4	13.8	
		4000	-	-	-	6.4	
		4200	−1.5	−11.6	−17	12.7	
5	BPF-3700T	3600	−55	-	-	-	50 MHz
		3650	−54.8	-	-	-	
		3700	−0.7	−22	−22.3	17.4	
		4000	-	-	-	4.8	
		4200	−0.58	−34	−31.5	11.1	

**Table 10:** Interference probability depending on protection distance and elevation angle of 5G-BS.

Distance Protection—km	5G-BS Elevation Angles (%)		
	15°	20°	30°
10	65.4	37.1	6.4
20	41.7	26.8	0.88
35	18.1	10.5	0.0

**Table 11:** MSE values for RBFNN and GRNN algorithms.

Protection Distance km	No. of Neurons	RBFNN MSE %	GRNN MSE %
10	0	0.168253	0.168258
	50	1.08857 × 10^−15^	1.08860 × 10^−15^
	100	4.17612 × 10^−18^	5.17622 × 10^−18^
**Elapsed time seconds**		**0.013071**	**0.438777**
20	0	0.128807	0.138813
	50	4.8190 × 10^−15^	4.8211 × 10^−15^
	100	2.81559 × 10^−17^	7.74620 × 10^−18^
**Elapsed time seconds**		**0.017157**	**0.400982**
35	0	0.168153	0.168270
	50	1.88574 × 10^−13^	1.89594 × 10^−13^
	100	3.61909 × 10^−18^	7.44741 × 10^−18^
**Elapsed time seconds**		**0.016235**	**0.471825**

## Data Availability

The researchers are required by copyright law to keep their data private.
